# Book Reviews

**Published:** 1892-01

**Authors:** 


### Book Reviews.

Ptomaines or Leucomaines. By Victor C. Vaughan, Ph. D., M. D., Professor of Hygiene and Physiological Chemistry in the University of Michigan and Director of the Hygienic Laboratory, and F. G. Novy, Sc. D., M. D., Assistant Professor of Hygiene and Physiological Chemistry in the University of Michigan. Lea Brothers & Co., Philadelphia.

This being the first work collecting and dealing with chemical factors in the causation of disease, it may well be considered the pioneer work in that line.

Emanating from so high authority and dealing with the intricate problems of the causation of disease, it cannot help but contain much that is interesting to the student of scientific medicine, and he who searches for the pathogenic causes of disease cannot fail to be profited by a study of its contents.

The authors endeavor to show that the "Mechanical Interference Theory" of Bacteriology is inadequate, and ably advocate the theory of alkaloidal developments as a cause of disease.

That bacteria are toxicogenic or non-toxicogenic according as their chemical products are toxic or non-toxic.

We are impressed by a brief review of the work that it is not so much a study of the morphology of microbes as a study of their toxic chemical products that will lead us into the light of the causation of disease. That the pathogenic quality of a germ depends upon its capacity to elaborate a chemical product of greater or less toxic properties.

The chemistry and chemical relations of bacterial products occupy a considerable part of the book, concluding with a chapter on Autogenous Disease, which is very interesting and instructive.

If one desires to be conversant with the latest views and discoveries in this line we would advise a study of this work.

R. R. K.

---

Pocket Medical Formulary. By W. M. Powell, M. D., author of "Essentials of Diseases of Children"; Attending Physician to Children's Clinic at University Hospital, Philadelphia, etc. Philadelphia. W. B. Saunders.

This formulary does not differ materially from others which have been compiled. All are mere collections of prescriptions which have been found valuable in given conditions, from time to time. This one, which is as good as any other, is supplemented with an appendix containing a posological table, formulæ and doses for hypodermic medication, a diet list for various diseases, etc.

